# Inequalities in the social determinants of health and Chagas disease transmission risk in indigenous and creole households in the Argentine Chaco

**DOI:** 10.1186/s13071-019-3444-5

**Published:** 2019-04-27

**Authors:** María del Pilar Fernández, María Sol Gaspe, Ricardo E. Gürtler

**Affiliations:** 1 Laboratorio de Eco-Epidemiología, Facultad de Ciencias Exactas y Naturales, Universidad de Buenos Aires, Ciudad Universitaria, C1428EHA Buenos Aires, Argentina; 2 Instituto de Ecología, Genética y Evolución de Buenos Aires, Consejo Nacional de Investigaciones Científicas y Técnicas, Ciudad Universitaria, C1428EHA Buenos Aires, Argentina; 3 Present Address: Earth Institute, Columbia University, New York, NY 10025 USA

**Keywords:** Socio-economic inequalities, Social vulnerability, *Trypanonoma cruzi*, *Triatoma infestans*, Neglected tropical diseases, Integrated vector control

## Abstract

**Background:**

The social determinants of health (SDHs) condition disease distribution and the ways they are handled. Socio-economic inequalities are closely linked to the occurrence of neglected tropical diseases, but empirical support is limited in the case of Chagas disease, caused by the protozoan *Trypanosoma cruzi*. Herein we assessed the relationship between key structural SDHs and the risk of *T. cruzi* vector-borne transmission in rural communities of the Argentine Chaco occupied by creoles and an indigenous group (Qom). We used multiple correspondence analysis to quantify the household-level socio-economic position (social vulnerability and assets indices), access to health and sanitation services, and domestic host availability. We identified the most vulnerable population subgroups by comparing their demographic profiles, mobility patterns and distribution of these summary indices, then assessed their spatial correlation and household-level effects on vector domiciliary indices as transmission risk surrogates.

**Results:**

Qom households had higher social vulnerability and fewer assets than creoles, as did local movers and migrant households compared with non-movers. We found significantly positive effects of social vulnerability and domestic host availability on infected *Triatoma infestans* abundance, after adjusting for ethnicity. Access to health and sanitation services had no effect on transmission risk. Only social vulnerability displayed significant global spatial autocorrelation up to 1 km. A hotspot of infected vectors overlapped with an aggregation of most vulnerable households.

**Conclusions:**

This synthetic approach to assess socio-economic related inequalities in transmission risk provides key information to guide targeted vector control actions, case detection and treatment of Chagas disease, towards sustainability of interventions and greater reduction of health inequalities.

**Electronic supplementary material:**

The online version of this article (10.1186/s13071-019-3444-5) contains supplementary material, which is available to authorized users.

## Background

The social determinants of health (SDHs) are social, economic and cultural factors that condition both disease distribution and the ways they are handled [1]. The links between these factors and health outcomes have been widely recognized since the 1990s and gained increasing prominence with the TDR/WHO Steering Committee on Social, Economic, and Behavioral Research (SEB) established in 2000 [1, 2]. However, the SDHs are still not fully integrated into public health policies, and they are often disregarded in biomedical research focused on disease control because they fall outside the scope of traditional healthcare systems [1]. To address this issue, the World Health Organization has developed a conceptual framework to act upon SDHs (CSDH), which draws on the concept that the social position of individuals and population groups is the main determinant of health inequalities within a community [3]. This social stratification translates into differential exposures to health-adverse conditions among individuals, differential consequences resulting from exposure (socio-economic or health outcomes), and/or differential capabilities to recover [3].

Neglected tropical diseases (NTDs) are a heterogeneous group of parasitic and bacterial diseases that disproportionately affect impoverished and under-represented minority groups. NTDs cause a high disease burden in low and middle income countries and an underappreciated burden in the Group of 20 (G20) Nations derived from their highly focal occurrence [1, 4–6]. Based on the CSDH framework, the SDHs of NTDs include socio-economic and demographic factors such as ethnicity, gender, occupation, educational level and income (i.e. structural determinants), which affect other factors more directly associated with disease exposure and outcome (i.e. intermediary determinants), particularly household and dwelling characteristics [3, 7–9].

Poverty is considered the main structural determinant of NTDs because of its association with living conditions and access to health services [4, 10, 11]. Understanding poverty as a dynamic and multidimensional process (as opposed to a merely lack of resources) requires introducing the concept of social vulnerability, which considers the “defenselessness, insecurity, and exposure to risks, shocks and stress” experienced by households [12]. This concept summarizes the multiple interrelated structural and intermediary determinants associated with the socio-economic position of individuals and groups in a population. However, in the context of low- and middle-income countries, socio-economic inequalities have been studied using surrogate indicators such as educational attainment and household ownership of assets [10], which at best partially capture the full complexity of poverty.

Chagas disease, caused by the kinetoplastid protozoan *Trypanosoma cruzi*, is among the most important NTDs in Latin America, and presents a disproportionately high disease burden on indigenous communities and poor rural peasants in the Gran Chaco eco-region extending over Argentina, Bolivia and Paraguay [6, 13, 14]. Although poverty has been long acknowledged as the main driver of Chagas disease risk [14–17], evidence of the effects of socio-economic inequalities is limited compared to other NTDs, as stated in a recent systematic review [10]. Only 4.3% of the 93 studies included in the review evaluated the effects of socio-economic position on Chagas disease. A literature search using PubMed and Google Scholar (29 September 2018) with the terms “poverty”, “social vulnerability” and “social determinants”, combined with “Chagas disease”, “neglected tropical diseases”, “*Triatoma*”, “*Rhodnius*” and “*Panstrongylus*” confirmed the paucity of studies specifically addressing the socio-economic inequalities in Chagas disease. We only found eight additional studies in which at least one component of the socio-economic status was related to either the risk of *T. cruzi* infection or house infestation prevalence (Additional file 1: Text S1).

The present study stems from a broader long-term research programme on the eco-epidemiology and control of Chagas disease in the municipality of Pampa del Indio, a highly endemic, mostly rural area of the Argentine Chaco where creoles and an indigenous people (Qom) live in structural poverty. In this region the seroprevalence of *T. cruzi* in indigenous peoples tends to exceed that of creoles [18–26]. Particularly in Pampa del Indio, house infestation rates with the main vector of Chagas disease, *Triatoma infestans*, were higher in Qom than in creole households [27–29] and dogs and cats from Qom households exhibited a higher *T. cruzi-*infection prevalence than those owned by creoles [30]. These differences coincided with more precarious living conditions in Qom households associated with house infestation: lower housing quality, higher household size and overcrowding, lower educational level and fewer livestock or poultry [27–29, 31]. However, the effects of socio-economic inequalities on the risk of vector-borne transmission were not assessed in an integrated manner, particularly within ethnic groups.

This study addresses the gap in our understanding of the combined effects of structural and intermediary SDHs on key vector indices closely associated with the risk of vector-borne transmission of *T. cruzi* [26, 32, 33]. We assessed socio-economic inequalities between creole and Qom households and within these groups in a well-defined rural section of Pampa del Indio in order to identify the most vulnerable groups by evaluating their demographic profiles, mobility and migration patterns, and access to health services. To quantify household socio-economic status we constructed a social vulnerability index using multiple correspondence analysis (MCA) to synthesize the multiple dimensions of poverty. This method has been widely used in the construction of socio-economic and demographic indices, especially in low and middle income countries [34–36]. We also analyzed the effects of social vulnerability, host availability (a key ecological factor) and access to health services on the risk of vector-borne transmission, and their spatial patterns. We hypothesized that social vulnerability was tightly associated with other SDHs and domestic vector indices related to parasite transmission.

## Methods

### Study area

This study was conducted in a rural section of Pampa del Indio municipality (25°55′S, 56°58′W), Chaco Province, Argentina, which encompassed 7 communities and 587 houses as of 2015 [31]. This section (here denominated Area III) is a historic settlement area of the Qom people [37]. The last insecticide spraying campaign targeting house infestation with *T. infestans* in Pampa del Indio municipality took place in 1997–1998.

The study area was subjected to a vector control and disease research programme initiated in 2008 with a follow-up period of 7 years as of 2015. In October 2008, 31.9% of the occupied houses were infested with *T. infestans*, mainly within human sleeping quarters, and virtually all (93.4%) were sprayed with insecticides [27]. During the 2008–2015 vector surveillance phase we conducted annual triatomine surveys and selectively sprayed with insecticide the few foci detected. This strategy reduced house infestation to < 1% during 2008–2012, and no infested house was found in 2015 [31].

Local houses usually included a domicile (i.e. an independent structure used as human sleeping quarters, also denominated “domestic premises”), a patio and other structures within the peridomestic area (kitchens, storerooms, latrines, corrals, chicken coops and chicken nests) (Figure S1 in [27]). Although housing quality remained precarious over the seven-year follow-up, the proportion of domiciles with mud walls and tarred-cardboard roof (as opposed to a tin roof) significantly decreased [31]. A household was defined as all the people who occupy a housing unit including related and nonrelated family members [38].

### Study design and household survey

This study complied with STROBE recommendations for observational studies [39], and the ethical principles included in the Declaration of Helsinki (Ethical Committee “Dr Carlos A. Barclay”, Protocol ref. TW-01-004).

All houses were registered and their location georeferenced with a GPS receiver (Garmin Legend; Garmin Ltd., Schaffhausen, Switzerland) in October 2008. The head of each household was informed of the purpose and protocol of the study, and gave oral consent. An environmental and socio-demographic survey was conducted as described elsewhere [27]. We collected information on the name of the head of each household, the number of residents by age class, the number of domestic animals of each type (dog, cats, poultry, goats, pigs, cows and equines) and their resting places, type and frequency of use of domestic insecticides, and the date of the last insecticide spraying conducted by vector control personnel or any other third party using manual compression sprayers. The ethnic group of the household was assigned on the basis of whether they spoke Qom language, participated in traditional Qom organizations, and took into account the tenants’ physical features and cultural practices. Multiethnic households (< 5%) (i.e. formed by at least one person self-identified as Qom and at least one person self-identified as creole) [40], were classified as Qom given their self-identification and cultural practices. The domiciles’ construction materials and other characteristics were registered, including refuge availability for triatomines, time since construction, and the area of the domicile. Refuge availability was determined visually by a skilled member of the research team and scored in one of five levels ranging from absence to very abundant refuges [28]; only the three top categories were actually observed in domiciles.

The recorded data were used to compute household-level surrogate indices for wealth, educational level and overcrowding as described elsewhere [27]. The goat-equivalent index represents a small stock unit that quantifies the household number of livestock (cows, pigs, goats) and poultry owned in terms of goat biomass. Household educational level was defined as the mean number of schooling years attained by household members aged 15 years-old (y.o.) or more. The overcrowding index was defined as the number of human occupants per sleeping quarter; the presence of 3 or more occupants per room was taken as critical overcrowding.

Each household’s location, demographic information and status was updated at each survey during the seven-year follow-up. The socio-demographic and environmental questionnaire was extended during the 2012–2015 surveys to include detailed information of each dweller and the use of personal protective practices (i.e. domestic insecticides and bednets). Although these protective practices were possibly used by householders to reduce the nuisance caused by blood-feeding insects and other domestic pests, they can exert an effect on reducing the exposure to triatomine vectors. We registered the name of each household resident, their relationship to the head of the household, age, gender, parents’ names, education and employment information, and whether they received some type of welfare support. Households were classified as encompassing one person only, one nuclear family (i.e. household consisting of at least one parent and their children), extended families (i.e. one nuclear family plus non-nuclear relatives, including more than one nuclear family), and other (non-family households and households consisting of second-degree relatives only).

The two censuses conducted in 2012 and 2015 allowed us to verify whether individual residents registered in 2012 were still residing in the same house in 2015 or had moved during the intervening period. We also registered any death, birth, and addition (and origin) of any new resident. This information was used to determine individual mobility during the 2012–2015 period: residents were classified as in-migrants or out-migrants (to or from outside the study area, respectively, including individuals coming from or leaving to a different section within Pampa del Indio municipality), and local movers (those who moved to a different house within Area III, i.e. local mobility). When the entire household out-migrated over this period, we asked their neighbors about their destination. Mobility at the household level (i.e. the mobility pattern of the household as a whole, as opposed to the mobility pattern of each member) was derived from individual mobility data and classified as: movers (i.e. households that changed its exact residential location within Area III), non-movers (i.e. households that remained at the same residential location), and migrant households (i.e. households that had in- or out-migrated from Area III) [31].

In 2015 we also collected information on access to health services and sanitary conditions: drinking water supply, sanitation services, fuel used for cooking, whether they used the local hospital, the local primary healthcare post or both, ambulance access, and whether a community healthcare agent visited the household. We determined the Euclidian distance (in km) between each house and different healthcare facilities using QGIS and the georeferenced locations. We also gathered information on assets owned by each household: television, radio, cell phone, freezer, fridge, bicycle, motorcycle and/or automobile.

### Demographic rates

The population growth rate (annual percentage change) was estimated for the 2008–2012 period (4.1 years) and for the 2012–2015 period (2.3 years) as follows:

$$\frac{{\Delta {\text{ Population during the period}}}}{\text{Mid-year population}} \times 100$$

The mid-year total population was estimated as the average between the 2012 and 2015 populations, multiplied by the duration of the period [41].

We calculated the general fertility rate (GFR), and the crude birth and crude mortality rates of the population residing in the study area over the 2012–2015 period. Births included children born after December 2012 (not registered in the 2012 census) whose parents resided at the study area at the date of birth and were registered in the census performed in April 2015. Deaths included only people that were registered in the 2012 census and died before April 2015. The population of women of childbearing age in Argentina encompasses those between 15 and 49 y.o. [42].

The GFR (person-years, PY) was estimated as:

$$\frac{{{\text{Number of births in 2012}}{-}2015}}{\text{Mid-year total population of women of childbearing age}} \times 1000;$$

and the crude birth and crude death rates were estimated as:

$$\frac{{{\text{Number of births (deaths) in 2012}}{-}2015}}{\text{Mid-year total population}} \times 1000;$$

We also estimated the net migration rate for the 2012–2015 period as:

$$\frac{{{\text{Migrant population during 2012}}{-}2015}}{\text{Mid-year population}} \times 1000$$

The migrant population was considered as the sum of in-migrants and out-migrants into and from the study area [41].

The local demographic indicators were compared to provincial (Chaco Province) and national vital statistics derived from the latest national census undertaken in Argentina [42].

### Socio-economic, health access and sanitation indices

We constructed two socio-economic indices measuring social vulnerability and assets, and a health access and sanitation index using multiple correspondence analysis (MCA) to summarize their multidimensionality. The social vulnerability index was constructed for the 2008 and 2015 surveys. The 2008 social vulnerability index included characteristics of the domiciles (refuge availability, presence of cardboard roofs and/or mud walls, time since house construction and domestic area), and household socio-economic and demographic characteristics (overcrowding, goat-equivalent index and educational level). The 2015 social vulnerability index additionally included the presence of dirt floors, the household number of welfare support payments received at the time of the survey, and the household number of salaried employees. The asset index was estimated for 2015 only and included the assets most commonly owned by local residents as detailed above.

The health access and sanitation index included relevant variables measured at household level in 2015: drinking-water supply (piped drinking water, borehole, tanker truck or dug well), sanitation facilities (pour-flush latrines, pit latrines or no sanitation facilities), distance to the nearest primary healthcare post and to the local hospital (located in Pampa del Indio town), and other variables related to health access as described above.

### Host availability index

Using the same approach described above for the socio-economic and sanitary indices, we constructed a host availability index in domiciles as of 2008 based on a preliminary analysis showing that the household abundance of domestic animal hosts was positively correlated with larger household size. This index summarized the number of potential domiciliary hosts of *T. infestans* (adult and child residents, total number of dogs, cats and chickens nesting indoors), and in the case of dogs and cats, whether they rested within or in the proximity of the domicile. The host availability index was introduced to account for a potential confounding effect when analyzing the effects of social vulnerability on vector indices.

### Vector indices as transmission surrogates

All triatomines collected at baseline were identified taxonomically and the individual infection status with *T. cruzi* was determined by microscope examination of feces [27] or by molecular diagnosis using kDNA-PCR [43], achieving a coverage of 60% of all infested houses.

The occurrence of domiciliary infestation with *T. infestans* was determined by the finding of at least one live triatomine (excluding eggs) through any of the vector collection methods used (i.e. timed-manual searches, during insecticide spraying operations, and householders’ bug collections). The relative abundance of domiciliary *T. infestans* was calculated only for infested houses as the number of live bugs collected by timed-manual searches per 15 min-person per site, as described [27]. The same procedures were used to determine the occurrence of at least one *T. cruzi*-infected *T. infestans* in the domicile and its relative abundance.

### Data analysis

Coverage of vector, socio-demographic and environmental surveys reached 95.6% (*n* = 390) of all occupied households enumerated in October 2008, 94.6% (*n* = 421) in November 2012 and 93.7% (*n* = 449) in April 2015. For analysis, we excluded houses that were closed and those in which householders refused to provide information. For each variable we checked whether the missing values were missing completely at random by building a dummy binary variable (missing and non-missing values) and analyzing the significance of the Spearman correlation coefficient with any another independent variable in the data set, as described elsewhere [27]. Most of the variables with missing values were missing completely at random, except for educational level and overcrowding in 2008, in which the missing data corresponded to households that had moved or out-migrated by 2012 (the year when these data were collected). Assuming similar conditions prevailed over 2012 and 2008, these variables were back-corrected to 2008 whenever possible [27].

Normality and homoscedasticity of continuous variables were tested by the Shapiro–Wilks test (normality), the Cook–Weisberg test (homoscedasticity) and other graphical methods (QQ plot and residuals *vs* fitted values scatterplot). For all proportions, 95% confidence intervals (95% CI) were estimated using the Agresti & Coull method if sample sizes were greater than 50, and the Wilson method for smaller sample sizes [44]. For medians, we report the interquartile range (IQR) [45]. Medians were preferred over means when continuous variables deviated significantly from a normal distribution. For bivariate analysis of categorical variables, we used Chi-square and Fisher’s exact tests depending on sample size and other assumptions. In the case of bivariate analysis comparing categorical and continuous variables, we used non-parametric tests (i.e. Mann–Whitney and Kruskal–Wallis) when the continuous variables did not fit a normal distribution. Correlations between continuous variables were evaluated by Spearman’s rank correlation coefficients.

The MCA used to construct the summary indices is a multivariate analysis that reduces the dimensionality of the covariance matrix in linear combinations of the original variables [46]. The first dimension captures most of the variance (inertia), and the score for each household (value of the dimension) can be used as a quantitative index [34]. For a better interpretation, the indices were considered as −Dimension 1. The different dimensions can also be assessed graphically using biplots, which allow a better understanding of how the variables are interrelated and their relative contribution to the score [47]. Because MCA requires all the variables to be categorical, numeric variables were categorized according to their quartile distribution. We used multiple linear regressions to assess variations in household-based indices by ethnic group and mobility status (i.e. non-movers, movers and migrants) adjusted by the community in which they were located.

We used generalized linear models (GLM) [48] to analyze the effect of the household’s ethnicity, mobility pattern and the community it was located (i.e. independent variables) on each of the indices constructed by MCA as dependent variables (socio-economic vulnerability, host availability and health access and sanitation indices). We also used GLM models to assess the household-level effects of these socio-demographic indices (i.e. independent variables) on the risk of vector-borne transmission of *T. cruzi*, adjusting for ethnicity and considering possible interactions between independent variables. The response variables were the occurrence and relative abundance of *T. infestans*, and the occurrence and relative abundance of *T. cruzi*-infected *T. infestans*. In the case of binary response variables (i.e. occurrence), we used logistic regression models with logit as the link function and the relative risk expressed as odds ratios (OR). When the response variable was vector abundance, we used negative binomial models with log as the link function and the relative risk expressed as incidence rate ratios (IRR). Negative binomial regression was preferred to Poisson regression given the overdispersed distributions [49]. All analysis were implemented in Stata v.14.2 [50] and R v.3.2.3 (*lme4* and *car* packages) [51].

### Spatial analysis

Global point pattern analysis (univariate and bivariate) were performed using the weighted K-function implemented in Programita [52]. Random labeling was selected to test the null hypothesis of random occurrence of events among the fixed spatial distribution of all houses. We used quantitative (abundance of infected vectors and the household social vulnerability and host availability scores) and qualitative labels (presence/absence of infected vectors) for each house (point). Monte Carlo simulations (*n* = 999) were performed and the 95% ‘confidence envelope’ was calculated with the 2.5% upper and lower simulations. Additionally, local spatial analysis on the abundance of (infected) vectors were performed using the G* statistic implemented in PPA [53]. The selected cell size was 200 m (assuming that each house had at least three neighbors at the minimum distance of analysis), and the maximum distance was set at 6 km (i.e. half of the dimension of the area). We created heatmaps (i.e. density maps) to visualize the spatial aggregation of the demographic and socio-economic indices using a kernel density estimation algorithm within a radius of 200 m as implemented in QGIS 2.18.11.

## Results

### Demographic profile

**Fig. 1:**
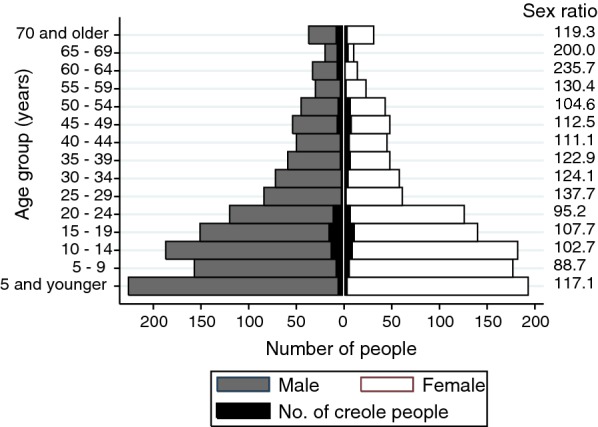
Age-sex pyramid and sex ratio per five-year age group in Area III of Pampa del Indio, Chaco, Argentina in 2015

The total population registered increased from 2392 people in 2008 to 2462 in 2012, and to 2548 in 2015. The demographic changes occurred more rapidly in the 2012–2015 period compared to 2008–2012: the annual population growth rate nearly doubled (1.5 *vs* 0.7%, respectively) and the proportion of creoles significantly decreased from 8.7 to 6.9% (*χ*^2^ = 3.8, *df* = 1, *P* = 0.05), while it had remained unchanged over 2008–2012 (*χ*^2^ = 0.1, *df* = 1, *P* = 0.8). The 2015 population showed a young age structure, whereby 43.8% of the total population were younger than 15 y.o. (Fig. 1, Table 1). The gender structure was biased towards males (110.9 males per 100 females), more evidently in children younger than 5 y.o. and in groups older than 25 y.o.

**Table 1:** Population and household characteristics by ethnic group registered in Area III of Pampa del Indio, Chaco, Argentina in 2015

Demographic characteristics	Qom	Creole	Total
Ethnic group (%)	93.1	6.9	100
Age structure			
Population < 15 y.o. (%)	45.4	22.7	43.8
Population ≥ 65 y.o. (%)	5.1	17.0	5.9
Median age	17.4	28.9	17.5
Ageing index^a^	11.2	75.0	13.6
Gender structure			
Females (%)	47.9	40.7	47.4
Males (%)	52.1	59.3	52.6
Sex ratio^b^	108.7	145.8	110.9
Demographic indicators			
Females of childbearing age (%)	20.8	22.7	20.9
Children per 100 females of childbearing age^c^	81.0	20.0	65.3
General fertility rate (per 1000 PY)			133.4
Crude birth rate (per 1000 PY)			30.5
Crude mortality rate (per 1000 PY)			4.2
Household characteristics			
Household size, median (IQR)	6 (4–8)	3 (1–5)	5 (3–8)
Overcrowding, median (IQR)	3.0 (2.0–5.0)	1.5 (1.0–2.5)	3.0 (2.0–4.5)
Household with children < 15 y.o. (%)	80.7	36.4	75.2
Household composition (%)			
One person only	7.0	28.0	9.7
Nuclear family	50.5	50.9	50.6
Extended family	40.4	15.8	37.2
Other^d^	2.1	5.3	2.5

^a^Adults (≥ 60 y.o.) per 100 children (< 15 y.o.)

^b^Number of males per 100 females

^c^Children < 5 y.o. per 100 females of childbearing age (15–49 y.o.)

^d^Two or more people with no familial ties

The population growth rate between 2012 and 2015 was mainly driven by high fertility and crude birth rates (Table 1). The local GFR (133.4 per 1000 PY) was 1.6 and 2.1 times higher than the fertility rate of Chaco Province (83.2 births per 1000 PY) and Argentina in 2010 (63.2 births per 1000 PY), respectively. The local crude birth rate (30.5 per 1000 PY) was 1.5 and 1.7 times higher than that of Chaco (19.9 per 1000 PY) and Argentina (17.7 per 1000 PY), respectively. In contrast, the crude mortality rate (4.2 per 1000 PY) was half of that estimated at province- and nation-wide levels (6.5 and 7.7 per 1000 PY, respectively).

The overall population structure was mainly driven by the Qom subgroup, which represented 93.1% of the local population, had a significantly lower median age than creoles (Kruskal-Wallis test, *P* < 0.001), and an eight-fold lower ageing index (*χ*^2^ = 67.4, *df* = 1, *P* < 0.001) (Table 1). Although the sex ratio, defined as the number of males per 100 females [38], was significantly higher in creoles (OR = 1.4, CI: 1.0–1.8, *P* = 0.05), the percentage of women of childbearing age was similar between Qom and creoles (Table 1). Nonetheless, the number of children per 100 women was 4 times higher for the Qom, and the proportion of Qom households with children < 15 y.o. approximately doubled that found among creole’s (Table 1). Household size and overcrowding were congruently two-fold higher among the Qom (Kruskal-Wallis test, *χ*^2^ = 40.7, *df* = 1, *P* < 0.001 and *χ*^2^ = 45.0, *df* = 1, *P* < 0.001, respectively). Household composition also varied between both groups (*χ*^2^ = 32.5, *df* = 3, *P* < 0.001): the frequency of extended families was 2.5 higher among the Qom, while creoles had a greater proportion of households composed of only one person (Table 1).

### Migration and mobility patterns

**Fig. 2:**
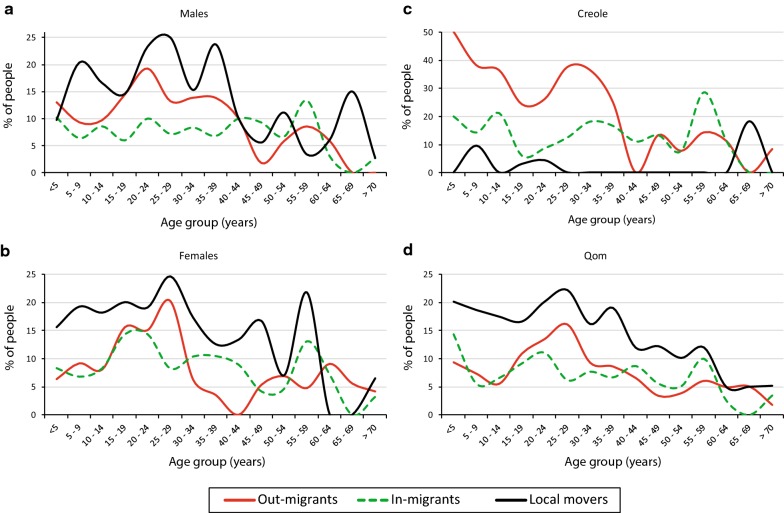
Migration and mobility patterns by age group, gender (**a**, **b**) and ethnic group (**c**, **d**) in Area III of Pampa del Indio, Chaco, Argentina, for the 2012–2015 period. Out-migrants and in-migrants refer to those who moved out or into the study area, respectively, regardless of whether they moved within the same municipality or to another town or city. Local movers changed their residential location within Area III

The overall impact of migration on population change between 2012 and 2015 was slightly negative (-37 people), with in-migration almost compensating out-migration. However, up to 15.4% of the population migrated (net migration rate was 82.8 per 1000 PY) during this period, and a similar proportion of the population (14.1%) changed residency within the area (i.e. local mobility). These patterns differed by age group and gender. Out-migration surpassed in-migration for age groups younger than 40 y.o. whereas this trend was reversed for older groups, which displayed a mostly positive net migration (Fig. 2a, b). Out-migration peaked in young adults, earlier in males (20–24 y.o.) than females (25–29 y.o.). The main reason for migrating (self-reported or by their relatives) was to start a new family (45%), followed by employment opportunities or educational reasons (19%). Unlike migration, local mobility within the area was sustained for all young age groups, and young adults were the most mobile (Fig. 2a, b). This pattern suggests that internal mobility also occurred at household level, involving young parents with their children. Nearly one in three (32.8%) of the in-migrants had been born in Area III and represented “return migrants”.

Migration and mobility patterns also varied significantly between ethnic groups (*χ*^2^ = 81.7, *df* = 3, *P* < 0.01) (Fig. 2c, d). Although non-movers represented the largest fraction of creoles (60.4%) and Qom (66.3%), the proportion of migrants within creoles was nearly two-fold greater than in the Qom population (36.5 *vs* 16.7%, respectively). Among creole migrants out-migration surpassed in-migration (Fig. 2c), whereas the overall net migration was almost nil for Qom people (8.6% out-migrants and 8.1% in-migrants). In contrast, local mobility was five-fold greater among Qoms rather than creoles (17 *vs* 3%, respectively).

Demographic and housing characteristics varied among non-movers, movers and migrant households (Additional file 2: Table S1). As of 2015, movers and migrant households occupied mud-built houses more frequently (91.5 and 83.3%, respectively) than non-movers (59%) (Fisher’s exact tests, *P* < 0.001), despite improvements in house quality compared to 2012. Although household size was not significantly associated with household mobility (Kruskal–Wallis test, *χ*^2^ = 1.6, *df* = 2, *P* = 0.4), movers and migrant households had significantly greater overcrowding and more frequent presence of children < 15 y.o. than non-movers (Kruskal–Wallis test, *χ*^2^ = 6.2, *df* = 2, *P* = 0.04). In agreement with individual-based mobility patterns, most movers and migrant households consisted of nuclear families.

### Host availability

The median household size in 2008 was 6 people (IQR = 4–8), including 2 children < 15 y.o. (IQR = 1–4), 3 dogs (IQR = 2–5), no cats, and 11 chickens (IQR = 2–28), for both ethnic groups (Chi-square tests, *P* > 0.1 in all cases) (Additional file 2: Table S2). Most houses (89%) had at least one dog, 40% had at least one cat and around 20% had chickens resting indoors. Householders reported that cats and dogs rested indoors or nearby (veranda or next to the outside wall) in 68% and 50% of houses, respectively; this was significantly more frequent in Qom than creole households (52.6 *vs* 29%; *χ*^2^ = 7.6, *df* = 1, *P* < 0.01). The host availability index derived from the MCA captured half of the total variability and reflected the gradient in host abundance (Additional file 3: Figure S1a); larger households were associated with a greater abundance of domestic animals associated with the domicile. The host availability index was significantly greater in Qom than creole households (Kruskal-Wallis test, *χ*^2^ = 13.5, *df* = 1, *P* < 0.001) and in non-movers households compared to movers and migrant households (Kruskal–Wallis test, *χ*^2^ = 8.7, *df* = 2, *P* = 0.01).

### Socio-economic profile

The demographic and socio-economic characteristics of Qom underperformed those from creole households both at baseline (2008) and over the 2012–2015 period (Table 2). Creole households inhabited larger and higher quality houses, with lower refuge availability (Fisher’s exact test, *P* < 0.001). Qom’s housing quality significantly improved between 2008 and 2012–2015 (Fisher’s exact test, *P* < 0.001). Qom households had greater overcrowding and lower educational level than creoles (Fisher’s exact test, *P* < 0.001 and *P* = 0.03, respectively). In general, formal employment was scarce, and the main economic activities and source of income were related to agricultural and husbandry practices for both ethnic groups. A significantly higher proportion of creole households based their livelihoods on agricultural or animal husbandry practices (Fisher’s exact test, *P* = 0.03) and had higher goat-equivalent indices than Qom households (Fisher’s exact test, *P* = 0.01), who were more dependent on welfare support (Fisher’s exact test, *P* = 0.01).

**Table 2:** Domicile construction characteristics, household socio-demographic characteristics and improved access to water and sanitation by ethnic group in Area III of Pampa del Indio, Chaco, Argentina, in 2008 and 2012–2015

Household-level factors	% of households (number of households)			
	Qom		Creole	
	2008	2012–2015	2008	2012–2015
Domicile characteristics				
High housing quality^a^	21 (337)	32 (447)	58 (51)	71.9 (64)
High refuge availability for *T. infestans*	70 (337)	76.5 (414)	43.1 (51)	56.6 (53)
Small domestic area (< 30 m^2^)	43.8 (292)	56.4 (385)	30.4 (46)	32.1 (56)
Household socio-demographic characteristics				
Critical overcrowding^b^	56.3 (231)	59.7 (367)	22.2 (45)	13.0 (54)
Low educational level^c^	58.0 (250)	59.9 (442)	43.5 (46)	45.3 (64)
Household economic activity and income				
Agriculture or animal husbandry	nr	79.8 (362)	nr	92.2 (51)
High goat-equivalent index^d^	12.2 (337)	9.4 (447)	59.2 (49)	57.8 (64)
At least one salaried employee	nr	14.1 (371)	nr	15.4 (51)
At least one welfare support	nr	77.6 (371)	nr	59.6 (51)
No source of income or economic activity	nr	6.6 (362)	nr	3.9 (51)
Assets				
Television	nr	61.5 (371)	nr	73.1 (52)
Fridge	nr	32.3 (371)	nr	53.8 (52)
Freezer	nr	26.4 (371)	nr	57.7 (52)
Radio	nr	53.1 (371)	nr	78.5 (52)
Cell phone	nr	51.7 (371)	nr	73.1 (52)
Bicycle	nr	43.4 (371)	nr	40.4 (52)
Motorcycle	nr	50.7 (371)	nr	61.5 (52)
Automobile	nr	2.2 (371)	nr	26.9 (52)
Sanitation services				
Pour-flush latrine^e^	nr	34.3 (350)	nr	56 (50)
Pit latrine	nr	54.9 (350)	nr	10.9 (50)
No sanitation facilities	nr	10.9 (350)	nr	8 (50)
Drinking-water supply				
Piped drinking water^f^	nr	56.5 (368)	nr	21.6 (51)
Tanker truck water	nr	27.1 (368)	nr	35.3 (51)
Dug well^f^	nr	9.8 (368)	nr	35.3 (51)
Borehole^f^	nr	6.5 (368)	nr	7.8 (51)
Fuel used for cooking				
Solid fuels in open fire or leaky stove	nr	44.7 (367)	nr	21.6 (51)
Natural gas	nr	33.2 (367)	nr	66.7 (51)
Both	nr	22.1 (367)	nr	11.8 (51)

^a^Brick walls, tin roofs and cement floors

^b^≥ 3 persons per sleeping quarter

^c^≤ 6 schooling years among household residents > 15 y.o

^d^> 30 goat-equivalents

^e^Improved sanitation facility

^f^Improved water source

*Abbreviation*: nr, not recorded

**Fig. 3:**
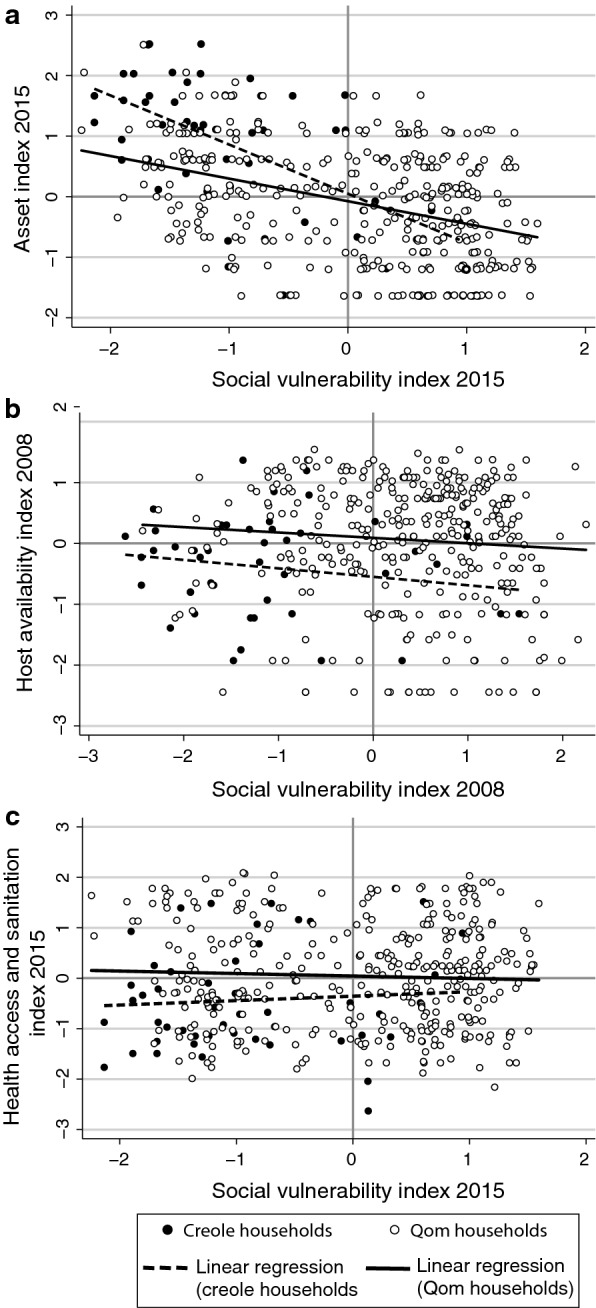
Household social vulnerability *vs* the asset index in 2015 (**a**), host availability in 2008 (**b**) and health access and sanitation index in 2015 (**c**), by ethnic group in Area III of Pampa del Indio, Chaco, Argentina

The social vulnerability index derived from the MCA captured 73.5% of the observed variability and the asset index captured 79.7%, summarizing socio-economic differences between and within ethnic groups (Additional file 3: Figure S1b, c). High social vulnerability indices were associated with smaller and more recently built houses having mud walls, cardboard roofs and dirt floors, overcrowded households, low educational level, low goat-equivalent index and lower income (including lower welfare support). The social vulnerability index was negatively and significantly correlated to the asset index in Qom (Spearmanʼs *ρ* = −0.4, *P* < 0.001) and creole households (*ρ* = −0.6, *P* < 0.001) (Fig. 3a). Multiple linear regression (*F*_(10,487)_ = 16.5, *P* < 0.001; adj *R*^2^ = 0.25; *n* = 498) showed that Qom households had higher social vulnerability than creoles’ (*β* = 0.8, *P* < 0.001), whereas movers and migrant households had higher social vulnerability than non-movers (*β* = 0.8, *P* < 0.001 for movers; *β* = 1, *P* < 0.001 for in-migrants; *β* = 0.7, *P* < 0.001 for out-migrants), after adjusting for the rural community in which they resided (related to distance to town). Social vulnerability indices for 2008 and 2015 were positively and highly significantly correlated both for Qom (Spearmanʼs *ρ* = 0.6, *P* < 0.001) and creole households (Spearmanʼs *ρ* = 0.8, *P* < 0.001). Host availability and social vulnerability indices were independent at the household level (Spearmanʼs *ρ* = −0.01, *P* = 0.8) (Fig. 3b).

### Access to health services and sanitary conditions

Very limited health services were available to the local communities as of 2015: they included a basic hospital located in town (up to 20 km away from the furthest community), and three primary healthcare posts located in each of the large communities, served by community health workers. The proportion of households with access to an improved water source (as defined by the WHO) in Area III (71.8%) was lower than the 2015 values reported [54] for Argentina (100%) and Latin America (89.4%) (Table 2). Similarly, access to a flush latrine was much lower in Area III (37.1%) than in Argentina (98.3%) and Latin America (76.9%). Qom households had significantly less access to improved sanitary services compared to creoles (Fisher’s exact test, *P* = 0.005) (Table 2). The overall access to an improved water source did not vary significantly between ethnic groups (Fisher’s exact test, *P* = 0.2), but the drinking water supply method did (Fisherʼs exact test, *P* < 0.001) (Table 2). Although more than half of Qom households had access to piped drinking water, 90% of them reportedly carried it from public standpipes and stored it in plastic containers.

The health access and sanitation index captured less than 50% of the overall variability among households (Additional file 3: Figure S1d). The most isolated households (further away from the local hospital and primary healthcare posts) had less access to piped water and made use of the nearest healthcare post more frequently than households closer to town, who made use of the local hospital more often. The health access index was independent of the social vulnerability in Qom (Spearmanʼs *ρ* = −0.01, *P* = 0.8) and creole households (Spearmanʼs *ρ* = 0.1, *P* = 0.5) (Fig. 3c). Multiple linear regression (*F*_(8,413)_ = 83; *P* < 0.001; adj *R*^2^ = 0.62; *n* = 422) showed that Qom households had higher access to improved water and sanitation services and health services than creoles (*β* = 0.3, *P* = 0.001), given their proximity to the hospital, primary healthcare post and piped water. These effects remained significant after adjusting for the rural community, but no significant effect of household mobility was detected.

### Socio-economic inequalities and vector-borne transmission

**Fig. 4:**
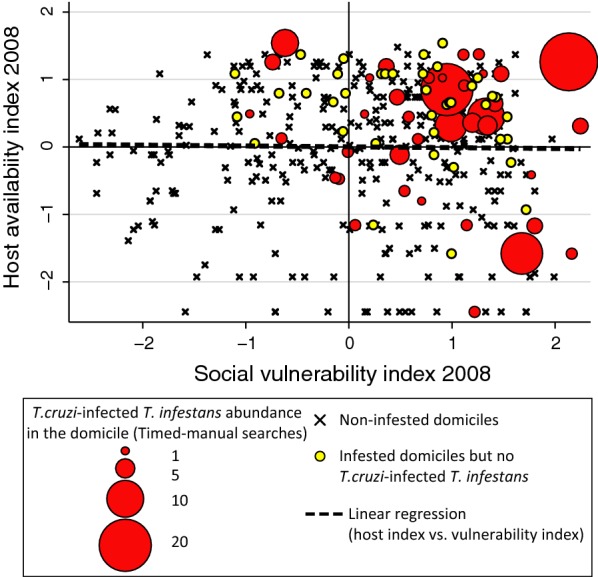
Household distribution according to host availability, social vulnerability and vector indices in 2008 prior to implementation of vector control and surveillance in Area III of Pampa del Indio, Chaco, Argentina

Baseline domiciliary infestation with *T. infestans* and vector abundance was significantly higher in more vulnerable households and in those with higher host availability; their interaction was non-significant (Table 3, Fig. 4). The relative abundance of *T. cruzi*-infected vectors also increased significantly with increasing household social vulnerability after adjusting for the host availability index (Table 3, Fig. 4). These effects remained significant after allowing for ethnicity. No significant effect of social vulnerability on the occurrence of at least one *T. cruzi*-infected vector was recorded, although a positive trend was evident.

**Table 3:** Multiple logistic regressions of domiciliary infestation with *T. infestans* and occurrence of *T. cruzi* infection, and negative binomial regressions of vector abundance and infected-vector abundance in domiciles in relation to household socio-economic and demographic characteristics in Area III of Pampa del Indio, Chaco, Argentina. (*n* = 77) at baseline

Model	Response variable	Domiciliary infestation		At least one infected vector^a^		Vector abundance^a^		Infected-vector abundance^a^	
		OR (95% CI)	*P*	OR (95% CI)	*P*	IRR (95% CI)	*P*	IRR (95% CI)	*P*
A	Social vulnerability index (2008)	1.9 (1.4–2.4)	< 0.001**	1.3 (0.7–2.4)	0.4	3.6 (1.6–3.3)	< 0.001**	2.0 (1.2–3.4)	< 0.01*
	Host availability index (2008)	1.5 (1.2–2.0)	0.001*	0.6 (0.4–1.2)	0.1	2.3 (1.6–3.3)	< 0.001**	1.4 (0.9–2.1)	0.1
B	Social vulnerability index (2008)	1.7 (1.3–2.4)	0.001*	1.7 (0.7–3.7)	0.2	3.0 (1.7–5.2)	< 0.001**	4.4 (1.8–10.9)	< 0.001*
	Host availability index (2008)	1.7 (1.2–2.4)	0.004*	0.7 (0.3–1.6)	0.4	2.8 (1.8–4.1)	< 0.001**	2.9 (1.3–6.4)	0.01*
	Health access and sanitation index (2015)	0.7 (0.5–0.9)	0.04*	2.0 (0.9–4.3)	0.1	1.0 (0.6–1.5)	0.9	1.5 (0.7–3.6)	0.3

^a^In the domicile

*Notes*: Model A included 386 households with infestation data and 77 households with vector infection data at baseline (2008). Model B included stable houses as of 2015 with infestation (*n* = 263) and vector infection data (*n* = 49)

**P < 0.001, *0.001 ≤ *P* ≤ 0.05

We additionally included the health access and sanitation index (as of 2015) in the model to evaluate its effect on pre-intervention vector indices. We found a negative association (*P* = 0.04) between health access and domestic infestation, indicating that houses that were closer to healthcare facilities had a lower probability of having been infested with *T. infestans* before vector control interventions (Table 3). No significant association was found between the health access index and the occurrence or abundance of *T. cruzi*-infected vectors.

Householders’ vector control and self-protection practices included insecticide use (mainly domestic aerosols) and bednets. Insecticide use was twice more likely among creoles than Qoms (85.7 *vs* 42.1%, respectively; *χ*^2^ = 32.6, *df* = 1, *P* < 0.001), whereas bednet use was 15 times greater (2.4 *vs* 35.2%) among the Qom (*χ*^2^ = 17.7, *df* = 1, *P* < 0.001). Insecticide use adjusted for ethnicity was much less frequent in households with greater social vulnerability (OR = 0.6, CI: 0.5–0.8, *P* < 0.001), whereas bednet use was significantly greater (OR = 1.7, CI: 1.2–2.4, *P* = 0.001). However, insecticide or bednet use did not exert any detectable effect on the abundance of infected vectors after adjusting for ethnicity and social vulnerability (OR = 0.7, CI: 0.2–1.6, *P* = 0.3 and OR = 3.0, CI: 0.8–10.6, *P* = 0.09, respectively).

### Spatial distribution of SDHs and transmission risk

**Fig. 5:**
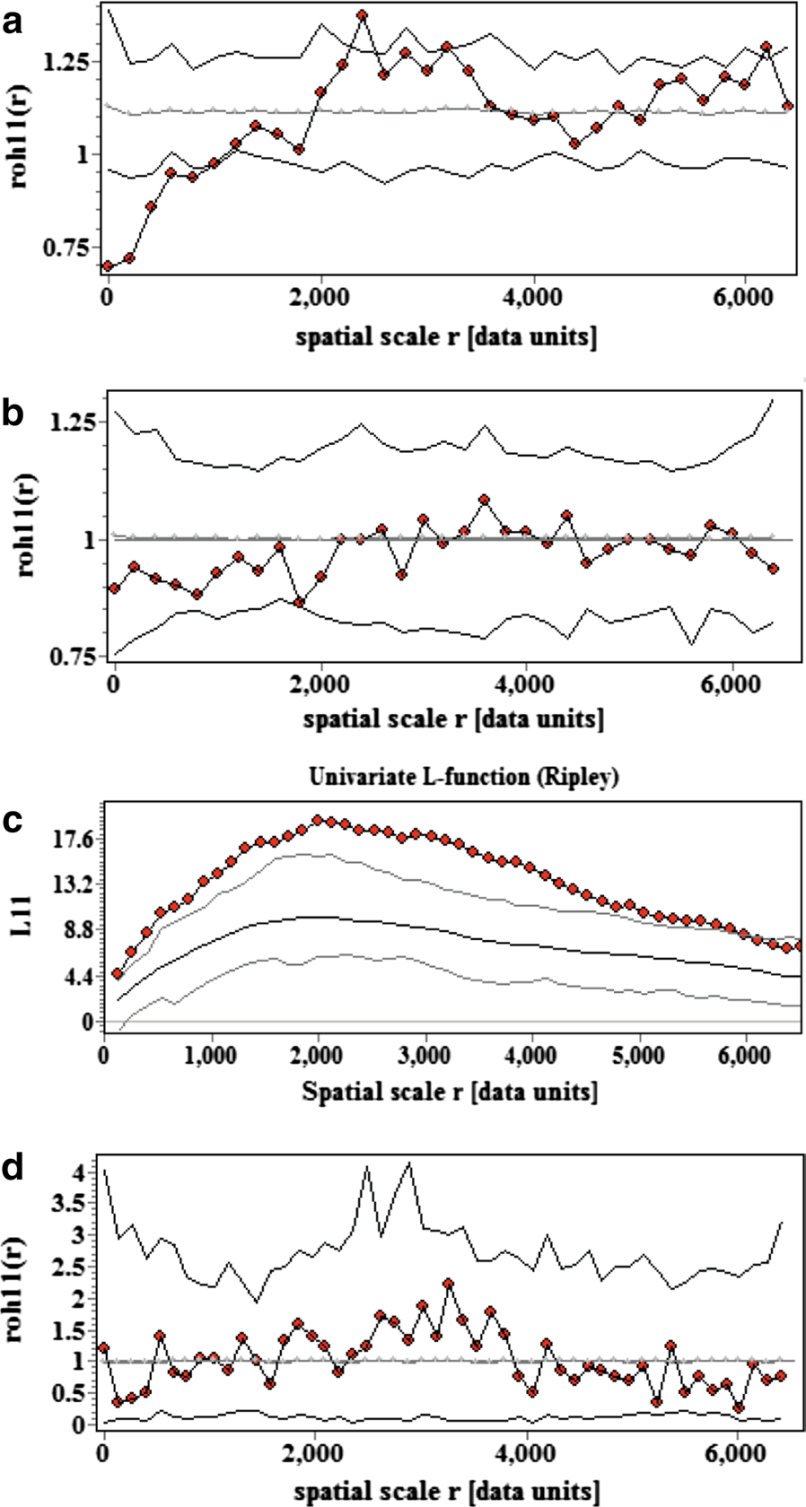
Global spatial analysis of quantitative and qualitative marks: social vulnerability (**a**), host availability (**b**), occurrence of at least one *T. cruzi*-infected *T. infestans* (**c**), and the relative abundance of infected vectors (**d**), area III of Pampa del Indio, Chaco, Argentina. The observed values correspond to the full dark circles and the lines correspond to the expected random pattern and its confidence envelopes

**Fig. 6:**
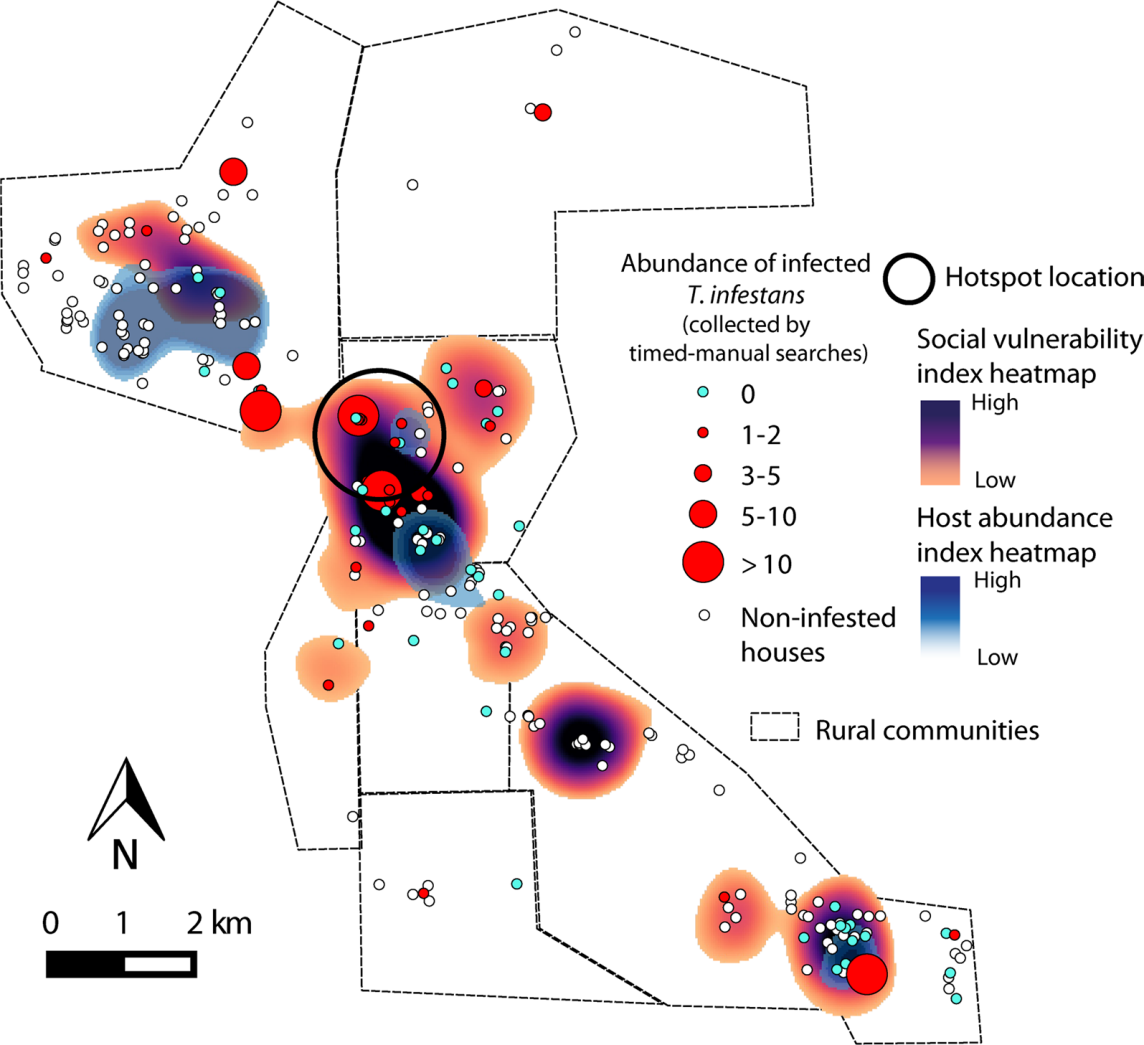
Heatmaps of the social vulnerability and host availability indices, and the relative abundance of *T. cruzi*-infected *T. infestans* in domicile at baseline (2008) in Area III of Pampa del Indio, Chaco, Argentina

The social vulnerability index at baseline (2008) displayed significant spatial autocorrelation up to 1 km, indicating that houses that were closer together had similar vulnerability (Fig. 5a), whereas the distribution of host availability did not differ significantly from a random spatial pattern (Fig. 5b). The occurrence of *T. cruzi*-infected *T. infestans* in the domicile was aggregated up to 6 km (Fig. 5c); this pattern ceased to be significant when only infested houses were considered. The relative abundance of infected vectors was not significantly aggregated (Fig. 5d). However, local spatial analysis revealed a hotspot of domiciliary infected vectors up to 1.8 km, which included 8 infested houses, 6 of which had at least one infected vector (Fig. 6). Although there was no significant spatial correlation between the abundance of infected vectors and social vulnerability or host availability indices (Additional file 4: Figure S2), most of the houses with infected vectors and the location of the hotspot coincided with the area where household vulnerability was higher (Fig. 6).

## Discussion

The social vulnerability index developed here revealed socio-economic inequalities between indigenous and creole households inhabiting a well-defined rural area in the Argentine Chaco, including household-level, within-ethnic group inequalities. By summarizing multiple SDHs associated with poverty, this quantitative index corroborated the direct association between household socio-economic position and the risk of vector-borne transmission of *T. cruzi*. To our knowledge, this synthetic approach has not been applied for Chagas disease or any other NTD. Socio-economic inequalities have often been analyzed under a reductionist approach, by which only selected aspects associated with socio-economic position were taken as independent variables within a broad set of putative factors, or as confounders of variables of interest [10]. The analysis of the association between social vulnerability, other SDHs and Chagas disease transmission risk indicates that: (i) social vulnerability and host availability had additive, positive effects on vector-borne transmission risk; (ii) household access to health and sanitation services was negatively associated with domestic infestation but not with social vulnerability or infected-vector abundance; and (iii) local movers and migrant households exhibited higher social vulnerability than non-movers.

We found empirical evidence supporting the role of poverty as the main structural SDHs of Chagas disease. The social vulnerability index summarized multiple SDHs related to poverty [7]: poor-quality housing, household overcrowding and low educational level, a subsistence economy, lack of formal employment, and dependence on welfare support. This index revealed variations between households and within demographic groups that would not be captured by income-based indices [36, 55], particularly in rural communities of the Gran Chaco where monetary income is principally dependent on scarce (informal and temporary) jobs and/or welfare support. The inverse correlation between social vulnerability and asset indices corroborates the close links between resource constraints and socio-demographic variables that reflect and perpetuate poverty (housing, education level, inadequate living conditions). Moreover, the positive correlation between the 2008 and 2015 social vulnerability indices indicated that the most vulnerable households at baseline continued to be the most vulnerable ones despite evident improvements in living conditions in the study area [31]. The concept of social vulnerability may be taken as an *ex-ante* risk that a household will fall below the poverty line, or if already poor, will remain in poverty [56]. When considered as a SDH, social vulnerability to disease(s) refers to a predisposition of certain individuals or groups to acquiring the disease(s) in question, and their capacity to respond to said disease(s) given their exposure, mobility capacity and resources to access the healthcare system [2, 57].

The higher social vulnerability of Qom compared to creole households is consistent with the higher infestation rates of Qom domiciles in Area III and elsewhere in Pampa del Indio [27–29]. In these multimodel-based analyses the effects of ethnic background ceased to be significant when other ecological and socio-economic variables more closely related to house infestation or vector abundance were allowed for [28]. The risk of preintervention house infestation increased with increasing refuge availability (closely related to building materials) and overcrowding, and decreased with increasing educational level and use of domestic insecticides [27]. Vector abundance was also positively associated with the household number of domestic animals and people [27–29], as in other areas infested with various triatomine species [58–61].

Instead of focusing on their independent effects, this study analyzed the combined effects of several variables related to housing construction and household socio-demographic variables (summarized in the social vulnerability index) to assess the overall impact of socio-economic position on the risk of vector-borne transmission. To this end, we used the abundance of *T. cruzi*-infected vectors because it is more closely related to the prevalence and incidence of human infection with *T. cruzi* [30, 32, 33, 62] than other indices, but the main outcomes with other indices (domiciliary infestation and vector abundance) were qualitatively congruent. In adjacent rural communities the relative risk of human infection increased almost three times with every infected vector collected in the domicile [26]. Households with both greater social vulnerability and host availability had the highest abundance of infected vectors, corroborating the occurrence of between- and within-group variations in transmission risks. Thus, our results support the key role of host availability as an ecological proximate factor [63], as in the Argentine Chaco, where vector abundance was closely related to domestic host abundance [27, 28, 60]. The host availability index summarizes the abundance of all possible domestic hosts and assumes that any of them may serve as a blood meal source.

The positive relationship between social vulnerability and infected-vector abundance is likely related to poor housing quality causing a large availability of refuges for triatomines, and to the householders’ type of prevention practices (or lack of them), since the use of domestic insecticides was positively associated with a better socio-economic position and purchasing power. In contrast, the use of bednets increased in the most vulnerable households, and both factors correlated positively with domestic vector abundance. Although cultural factors related to ethnicity may explain in part the increased use of bednets among the Qom, within-group differences also point towards the perceived risks of transmission and/or nuisance caused by high abundance of blood-sucking insects, including triatomines, as reported for malaria transmission in Africa [64]. Evidence of the negative association between the socio-economic position and infected-vector abundance at a household level, was also found at a higher scale when comparing their spatial distribution within the study area. As stated by Houweling et al. [10], “spatial clustering of infection because of geographic conditions, among other causes, is typical for most NTDs,” but it may also be context-specific, depending on the intersection between the social and ecological factors at play. Although the spatial analysis did not indicate a global spatial correlation between social vulnerability and the abundance of infected vectors, the hotspot of infected vectors overlapped with the most vulnerable households. Therefore, the contribution of social vulnerability to the spatial heterogeneity of transmission risk apparently exceeded the contribution of host availability, which showed a random spatial distribution.

The health access index was not associated with social vulnerability at the household level and creole households had a lower health access index than Qom households. The latter summarized various intermediary SDHs such as distance to healthcare facilities, access to improved water and sanitation services, and other health services, but it captured less than 50% of the variability between households and mostly reflected distance to the town. Households that were closer to town had greater access to health and sanitary services, which explains why creoles had lower access as their homes tended to be further away. The distance to healthcare facilities can be compensated by owning motor vehicles (26.9% of creole *versus* 2.2% of Qom households owned them), adding to the complex relationship between access and effective use of health services. Other studies have used travel time instead of Euclidian distance to address measure accessibility, but evidence of the association between socio-economic status and accessibility is scattered and seems to be context-dependent [65, 66]. Moreover, the use of health services by indigenous residents is frequently hindered by alleged discriminatory behaviors within the health system [67]. Indigenous community health workers have improved access to health care within the local Qom communities, but they are not involved in vector control actions. Although domestic infestation was significantly lower in houses with greater access to health services (but not infected-vector abundance), this may reflect the aggregation of non-infested, newly-built houses around health posts or their improved access to insecticides or capacity to demand vector control actions given their proximity to town.

The demographic indicators suggest the local communities were in the second stage of the demographic transition (moderate transition), characterized by elevated poverty levels, birth rates and young people, decreased mortality rates, and mainly occupying rural environments with deficient access to social services [41, 68]. This pattern in Pampa del Indio largely differs from department-, province- and nation-level indicators [42], reflecting the socio-demographic heterogeneities that characterize median-income and Latin American countries [4, 69].

The between-group differences in their demographic features were mostly related to local mobility and migration patterns. Young adults had the greatest mobility, with 42–50% changing residential location during a 28-month period, which is one of the regularities most frequently observed [70]. However, creole migration patterns reflected the traditional rural-to-urban movement, which began in the 1950s [71] and explains the ageing age-structure of local creoles. Qom migration patterns were much more complex: their migration rates equaled internal mobility, while in-migration almost fully compensated out-migration. Qom’s mobility is enhanced by a combination of socio-economic and cultural factors: nomadic traditions [72], formation of new families, household mobility to gain increased access to basic services (e.g. improved water sources and school), and cultural reasons (death of the head of family).

These patterns of local mobility explain the elevated housing turnover rate in the study area, which affects house infestation [27, 31] and can also determine heterogeneities in human-vector contact rates [73]. Of particular interest is the association between household socio-economic position, mobility and migration patterns. Migrant households and local movers had increased social vulnerability, and greater chances of occupying an infested house before and after interventions than non-movers [31], which in turn would increase their risk of exposure to *T. cruzi*-infected vectors.

Some limitations generated from the type and source of the demographic data need to be considered. Although many of the variables were registered by direct observation by one member of the research team, self-reported variables may be affected by an information bias. The language barrier in some Qom households may have enhanced this potential bias despite our careful attention to re-questioning any response that gave way for doubt while avoiding yes/no questions. The information bias for children aged 0–5 years is well known [41]; they are frequently underreported. The high rates of household and individual mobility presented some challenges when collecting census data: under-reporting may have occurred in the case of people who lived in the area over 2012–2015 but were absent at the time of the surveys, their houses were closed or they refused to participate. We may have also missed newborn children that moved out after birth and were no longer present in 2015, and deaths of those who had moved into the area after the 2012 census and died before the 2015 census. Therefore, both deaths and births were likely underestimated, and the demographic indices calculated here are the best approximation possible given the absence of more accurate demographic data. Other limitations related to vector indices have been discussed elsewhere [27].

## Conclusions

This study developed an integrative approach to focus on the household socio-economic position, one of the main structural SDHs, and its association with other SDHs and vector indices closely related to parasite transmission. This approach identified the groups that were most at risk within apparently uniformly impoverished rural communities and revealed that households with higher social vulnerability were at higher risks of exposure to infected vectors, and presumably, of becoming infected with *T. cruzi*. Such differentials will increase health inequalities and keep the affected individuals and demographic groups in a poverty trap [8, 15, 74]. The social vulnerability index may be adapted to identify the most vulnerable households affected by multiple health burdens. Most of the SDHs considered in this study lie outside traditional public health policies. There is a need to develop new sets of interventions and a new ways to implement public health programmes [75]. Although the approach proposed here can be applied more broadly, the association between the different SDHs may be context-specific and there is no universal protocol of intervention with respect to the determinants of health of NTDs [8]. Intervention strategies oriented to reduce the impact of SDHs must be tailored to specific social contexts, capacities and resources available, in order to maximize their impact and cost-effectiveness. The synthetic approach used here to assess socio-economic inequalities provides key information to tailor and guide targeted vector control actions, case detection and treatment of Chagas disease, and facilitate the integration with other health burdens, towards sustainability of interventions and greater reduction of health inequalities.

## Additional files

**Additional file 1: Text S1.** Summary of evidence on the association between Chagas disease risk and socio-economic aspects based on a literature search.

**Additional file 2: Table S1.** Household characteristics by household mobility and migrant condition patterns registered in Area III of Pampa del Indio, Chaco, Argentina between 2012 and 2015. **Table S2.** Household size and domestic animal abundance by ethnic group at baseline (2008) in Area III of Pampa del Indio, Chaco, Argentina.

**Additional file 3: Figure S1.** Biplots of the multiple correspondence analysis of host abundance in domiciles in 2008 (**a**), household socio-economic characteristics in 2015 (**b**), and health access and sanitation index in 2015 (**c**) in Pampa del Indio, Chaco, Argentina.

**Additional file 4: Figure S2.** Bivariate spatial analysis of the relative abundance of *T. cruzi*-infected *T. infestans vs* the social vulnerability (**a**) or host availability indices (**b**), Area III of Pampa del Indio, Chaco, Argentina. The observed values correspond to the full dark circles and the lines correspond to the expected random pattern and its confidence envelopes.

## Abbreviations

TDR/WHO: Special Programme for Research and Training in Tropical Diseases, World Health Organization
SDHs: social determinants of health
CSDH: Conceptual Framework to act Upon SDHs
NTDs: neglected tropical diseases
GFR: general fertility rate
PY: person-years
IQR: interquartile range
MCA: multiple correspondence analysis
