# Supplementary material for: Inequalities in the social determinants of health and Chagas disease transmission risk in indigenous and creole households in the Argentine Chaco
Source: Parasit Vectors. 2019 Apr 27;12:184. doi: 10.1186/s13071-019-3444-5 (PMC6487000; doi:10.1186/s13071-019-3444-5)
Supplement: Supplementary file 1 — Additional file 1: Text S1. Summary of evidence on the association between Chagas disease risk and socio-economic aspects based on a literature search. [file 13071_2019_3444_MOESM1_ESM.docx]

**Additional file 1: Text S1**

**Summary of evidence on the association between Chagas disease risk and socio-economic aspects**

Based on a literature review, we summarize the evidence found between Chagas disease and/or house infestation with triatomines and aspects related to socio-economic status. In addition to the Meta-analysis conducted by Houweling et al (2016), we expanded our search to include house infestation as an outcome.

Meta-analysis conducted by Houweling et al (2016)

Their question of interest was: What is the distribution within countries, by socioeconomic position, of LF, onchocerciasis, schistosomiasis, STH, trachoma, Chagas’ disease, HAT, leprosy, and VL?

The following references were found regarding Chagas disease.

1. Llovet, I., Dinardi, G. & De Maio, F. G. Mitigating social and health inequities: Community participation and Chagas disease in rural Argentina. *Glob. Public Health* **6,** 371–384 (2011).
2. Borges-Pereira, J., de Castro, J. A. F., da Silva, A. G., Zauza, P. L., Bulhões, T. P., Gonçalves, M. E., de Almeida, E. S., do Amparo Salmito, M., Pereira, L. R. M., Alves Filho, F. I., Correia-Lima, F. G. & Coura, J. R. Seroprevalence of Chagas disease infection in the State of Piauí, 2002. *Rev. Soc. Bras. Med. Trop.* **39,** 530–9
3. Pinto, F. S., Andrade, G. M. Q. de, Januario, J. N., Maia, M. C. A., Gontijo, E. D., Pinto, F. S., Andrade, G. M. Q. de, Januario, J. N., Maia, M. C. A. & Gontijo, E. D. Epidemiological profile of Trypanosoma cruzi-infected mothers and live birth conditions in the state of Minas Gerais, Brazil. *Rev. Soc. Bras. Med. Trop.* **46,** 196–199 (2013).
4. Cucunubá, Z. M., Flórez, A. C., Cárdenas, Á., Pavía, P., Montilla, M., Aldana, R., Villamizar, K., Ríos, L. C., Nicholls, R. S. & Puerta, C. J. Prevalence and risk factors for chagas disease in pregnant women in Casanare, Colombia. *Am. J. Trop. Med. Hyg.* **87,** 837–842 (2012).

Outcome: Chagas disease

1. Salas Clavijo, N. a., Postigo, J. R., Schneider, D., Santalla, J. a., Brutus, L. & Chippaux, J. P. Prevalence of Chagas disease in pregnant women and incidence of congenital transmission in Santa Cruz de la Sierra, Bolivia. *Acta Trop.* **124,** 87–91 (2012).
2. Cardinal, M. V., Sartor, P. A., Gaspe, M. S., Enriquez, G. F., Colaianni, I. & Gürtler, R. E. High levels of human infection with Trypanosoma cruzi associated with the domestic density of infected vectors and hosts in a rural area of northeastern Argentina. *Parasit. Vectors* **11,** 492 (2018).
3. Fung, H. L., Calzada, J., Saldaña, A., Santamaria, A. M., Pineda, V., Gonzalez, K., Chaves, L. F., Garner, B. & Gottdenker, N. Domestic dog health worsens with socio-economic deprivation of their home communities. *Acta Trop.* **135C,** 67–74 (2014).
4. Arrom-Suhurt, C. M., Arrom-Suhurt, C. H., Arrom-Suhurt, M. A., Rolón, M., Vega-Gómez, M. C. & Rojas de Arias, A. Socioeconomic profile and perceptions of Chagas disease in indigenous communities of the Paraguayan Chaco. *J. Public Health (Bangkok).* 1–10 (2018). doi:10.1007/s10389-018-1001-x

Outcome: House infestation

1. Ramsey, J. M., Alvear, A. L., Ordoñez, R., Muñoz, G., Garcia, A., Lopez, R. & Leyva, R. Risk factors associated with house infestation by the Chagas disease vector Triatoma pallidipennis in Cuernavaca metropolitan area, Mexico. *Med. Vet. Entomol.* **19,** 219–28 (2005).
2. Bustamante, D. M., De Urioste-Stone, S. M., Juárez, J. G. & Pennington, P. M. Ecological, social and biological risk factors for continued Trypanosoma cruzi transmission by Triatoma dimidiata in Guatemala. *PLoS One* **9,** e104599 (2014).
3. Gaspe, M. S., Provecho, Y. M., Cardinal, M. V., Fernández, M. P. & Gürtler, R. E. Ecological and sociodemographic determinants of house infestation by Triatoma infestans in indigenous communities of the Argentine Chaco. *PLoS Negl. Trop. Dis.* **9,** e0003614 (2015).
4. Dumonteil, E., Nouvellet, P., Rosecrans, K., Ramirez-Sierra, M. J., Gamboa-León, R., Cruz-Chan, V., Rosado-Vallado, M. & Gourbière, S. Eco-bio-social determinants for house infestation by non-domiciliated Triatoma dimidiata in the Yucatan Peninsula, Mexico. *PLoS Negl. Trop. Dis.* **7,** e2466 (2013).
