# Supplementary material for: Inequalities in the social determinants of health and Chagas disease transmission risk in indigenous and creole households in the Argentine Chaco
Source: Parasit Vectors. 2019 Apr 27;12:184. doi: 10.1186/s13071-019-3444-5 (PMC6487000; doi:10.1186/s13071-019-3444-5)
Supplement: Supplementary file 2 — Additional file 2: Table S1. Household characteristics by household mobility and migrant condition patterns registered in Area III of Pampa del Indio, Chaco, Argentina between 2012 and 2015. Table S2. Household size and domestic animal abundance by ethnic group at baseline (2008) in Area III of Pampa del Indio, Chaco, Argentina. [file 13071_2019_3444_MOESM2_ESM.docx]

**Additional file 2**

**Table S1.** Household characteristics by household mobility and migrant condition patterns registered in Area III of Pampa del Indio, Chaco, Argentina between 2012 and 2015.

|  | Household mobility | | |
| --- | --- | --- | --- |
| Household attribute | Non-movers | Movers | Migrant |
| Total number of households | 330 | 51 | 21* |
| Ethnic group (%) |  |  |  |
| Qom | 84.8 | 98.0 | 76.2** |
| Creole | 15.2 | 2.0 | 23.8 |
| Inhabiting mud houses in 2012 (%) | 59.6 | 91.5 | 83.3 |
| Inhabiting mud houses in 2015 (%) | 50.4 | 76.1 | 73.3 |
| Household size (median, IQR) | 6 (3-8) | 6 (4-7) | 5 (4-7) |
| Overcrowding (median, IQR) | 3.0 (1.7-4.5) | 3.3 (2.3-5) | 4.0 (1.8-7) |
| Size of nuclear family (median, IQR) | 4 (3-5) | 4 (3-6) | 4.5 (3.5-6) |
| Households with children <15 y.o. (%) | 73.0 | 84.3 | 76.2 |
| Type of household (%) |  |  |  |
| One person only | 10.3 | 5.9 | 19.0 |
| Nuclear family | 44.8 | 62.8 | 62.0 |
| Extended family | 42.7 | 28.8 | 19.0 |
| OtherϮ | 2.2 | 2.5 | 0.0 |
| * In-migrant households in 2015 only  ** Out-migrant households in 2012  Ϯ Two or more people with no familial ties | | | |

**Table S2.** Household size and domestic animal abundance by ethnic group at baseline (2008) in Area III of Pampa del Indio, Chaco, Argentina.

|  | **Ethnic group** | | |
| --- | --- | --- | --- |
|  | **Creole** | **Qom** | **Total** |
| **Number of households** | 40 | 346 | 386 |
| **Household size (%)** |  |  |  |
| 1-2 | 25.0 | 14.7 | 15.8 |
| 3-6 | 62.5 | 37.6 | 40.2 |
| 7-10 | 12.5 | 36.7 | 34.2 |
| >10 | 0.0 | 11.0 | 9.8 |
| **Number of children <15 y.o. (%)** |  |  |  |
| 0 | 40.0 | 21.4 | 23.3 |
| 1-2 | 37.5 | 25.7 | 26.9 |
| 3-4 | 15.0 | 27.2 | 25.9 |
| >4 | 7.5 | 25.7 | 23.8 |
| **Number of dogs (%)** |  |  |  |
| 0 | 5.0 | 11.6 | 10.9 |
| 1-2 | 27.5 | 27.2 | 27.2 |
| 3-4 | 35.0 | 34.1 | 34.2 |
| >4 | 32.5 | 27.2 | 27.2 |
| **Number of cats (%)** |  |  |  |
| 0 | 45.0 | 52.9 | 52.1 |
| 1-2 | 42.5 | 39.9 | 40.2 |
| >2 | 12.5 | 7.3 | 7.8 |
| **Number of chickens (%)** |  |  |  |
| 0 | 24.6 | 11.8 | 22.9 |
| 1-5 | 13.9 | 2.0 | 12.3 |
| 6-10 | 13.9 | 5.9 | 12.9 |
| 11-15 | 8.9 | 7.8 | 8.7 |
| >15 | 38.8 | 72.5 | 43.2 |
| **Number of chickens resting indoors (%)** |  |  |  |
| 0 | 77.5 | 77.2 | 77.2 |
| 1-2 | 10.0 | 11.8 | 11.7 |
| 3-9 | 7.5 | 6.1 | 6.2 |
| >10 | 5.0 | 4.9 | 4.9 |
