# Supplementary figures and images for: Inequalities in the social determinants of health and Chagas disease transmission risk in indigenous and creole households in the Argentine Chaco
Source: Parasit Vectors. 2019 Apr 27;12:184. doi: 10.1186/s13071-019-3444-5 (PMC6487000; doi:10.1186/s13071-019-3444-5)

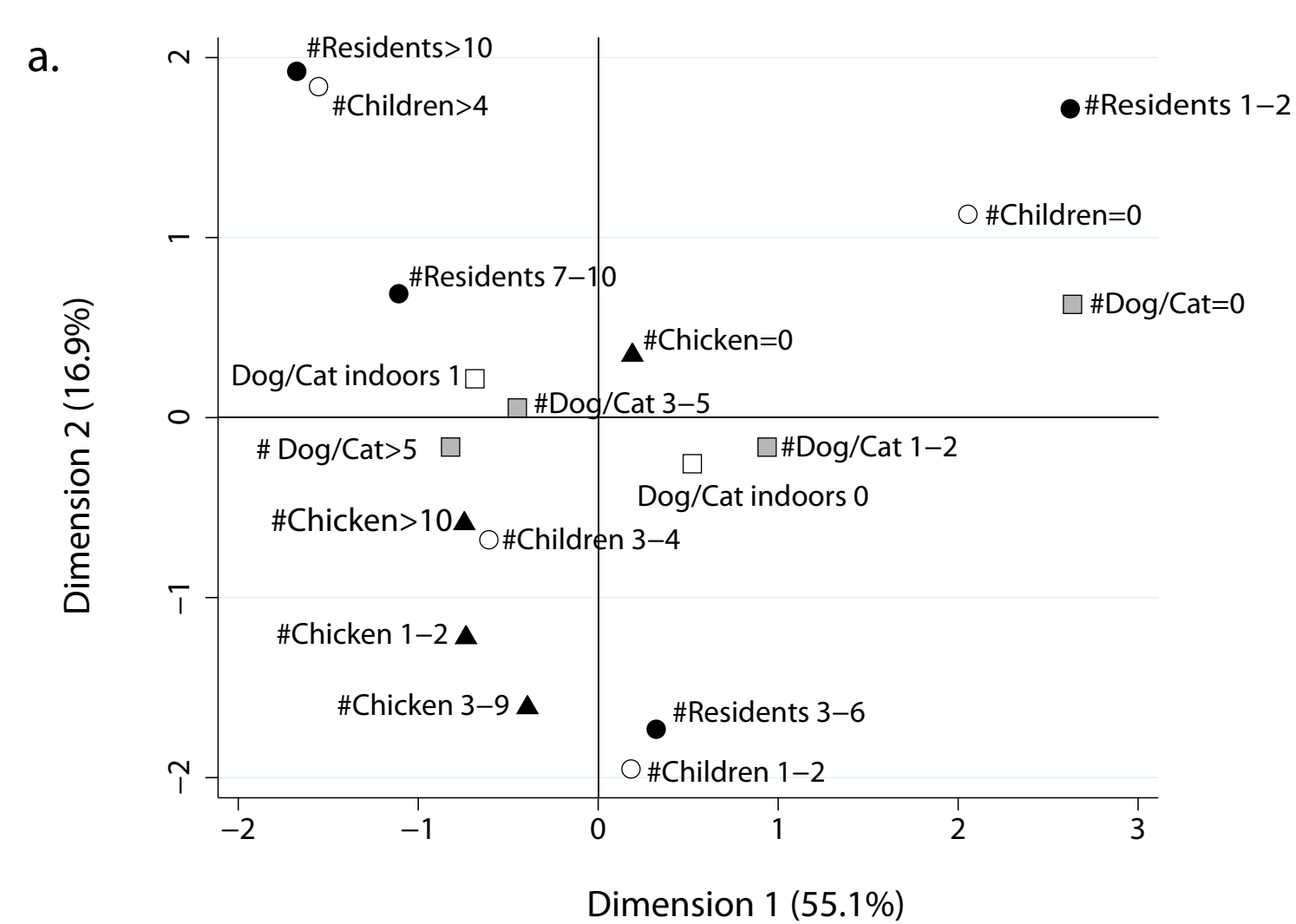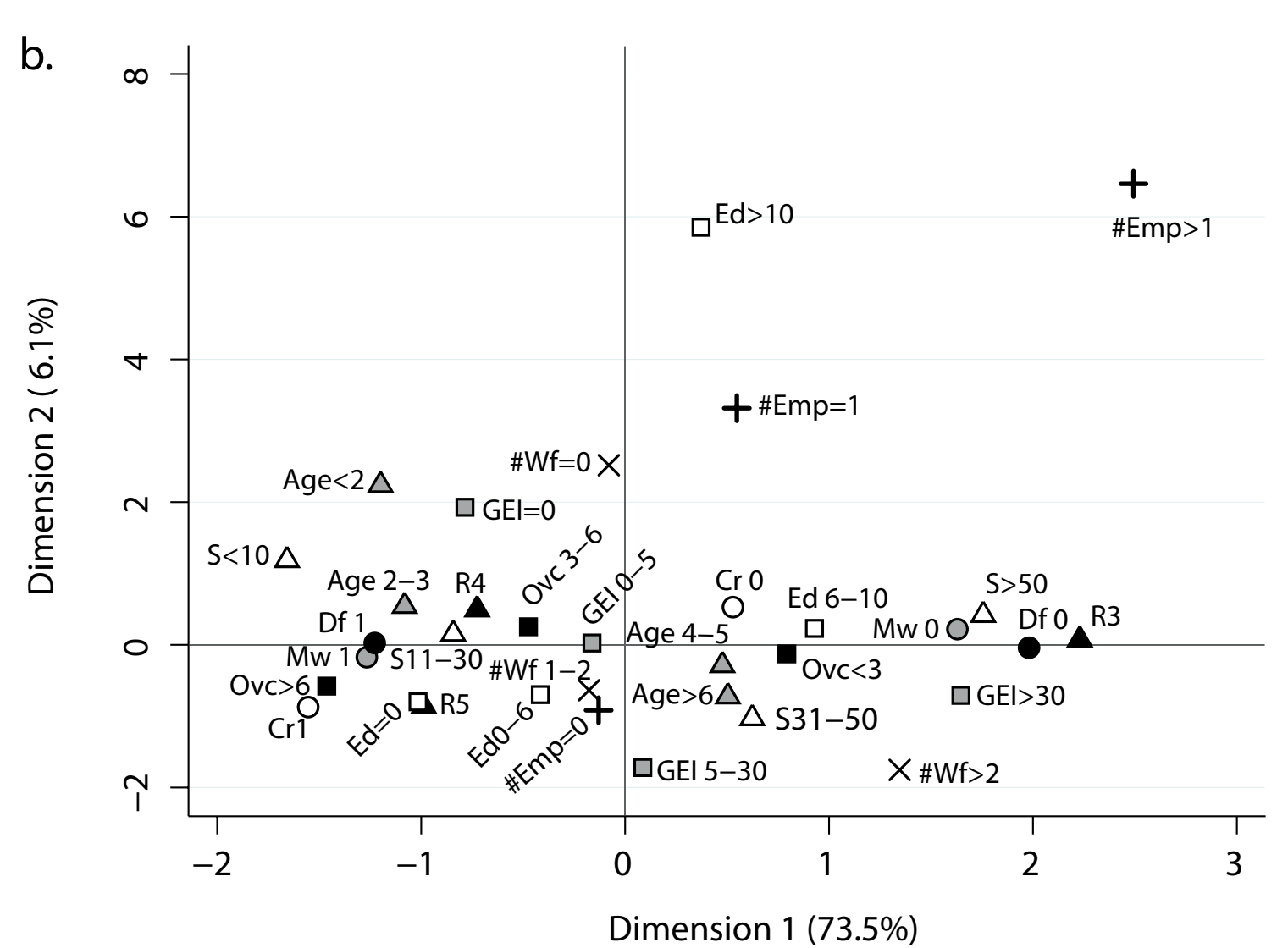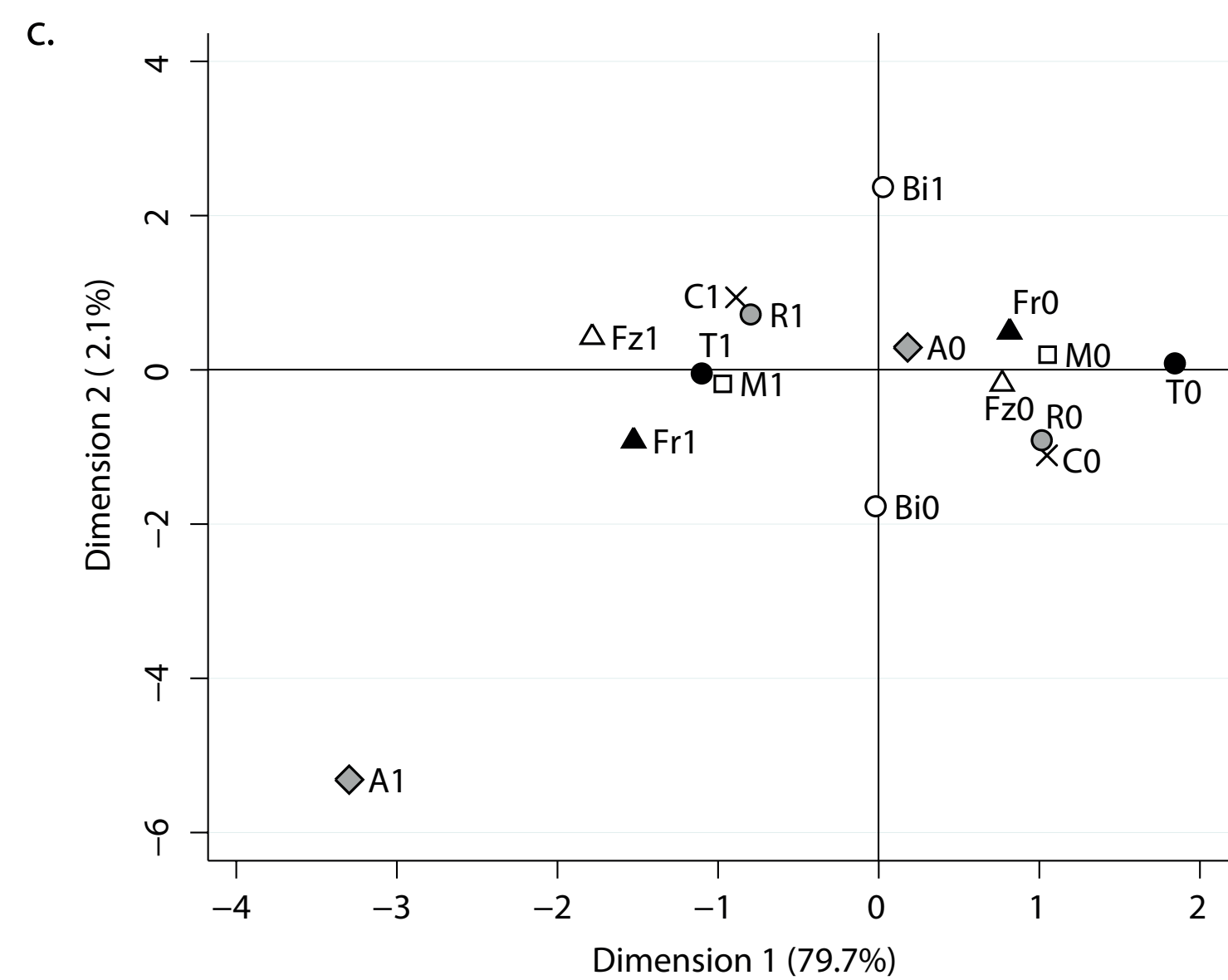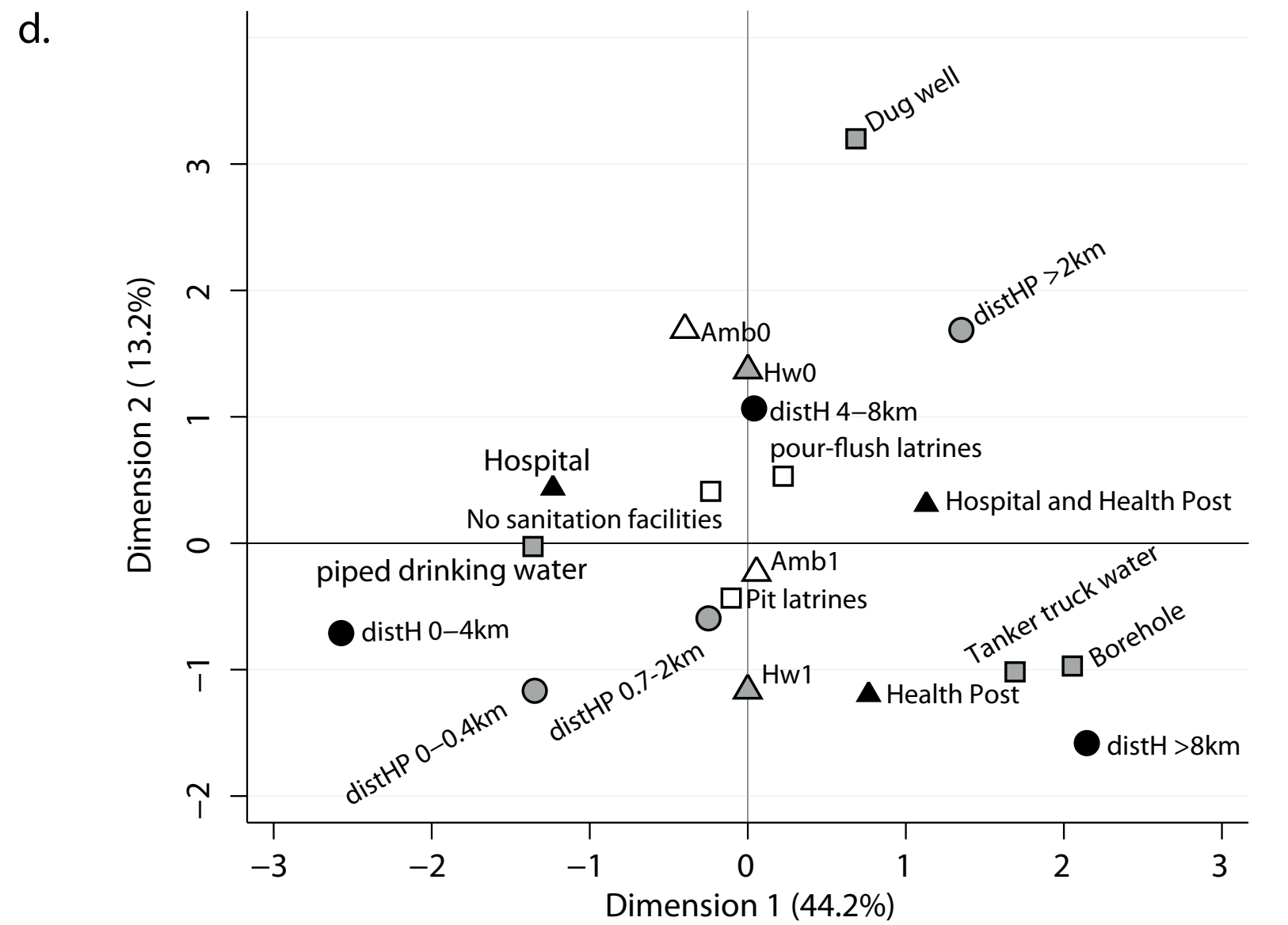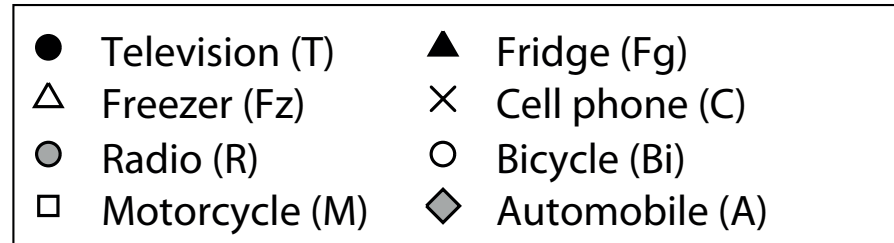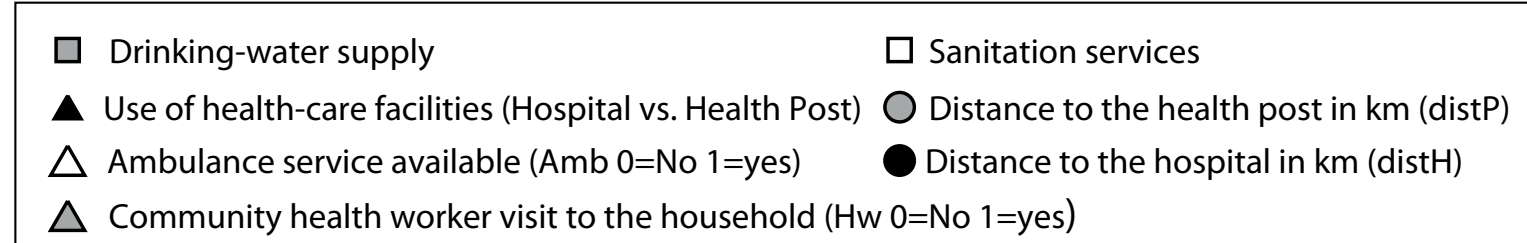

Supplement: Supplementary file 3 — Additional file 3: Figure S1. Biplots of the multiple correspondence analysis of host abundance in domiciles in 2008 (a), household socio-economic characteristics in 2015 (b), and health access and sanitation index in 2015 (c) in Pampa del Indio, Chaco, Argentina. [file 13071_2019_3444_MOESM3_ESM.pdf]

a.

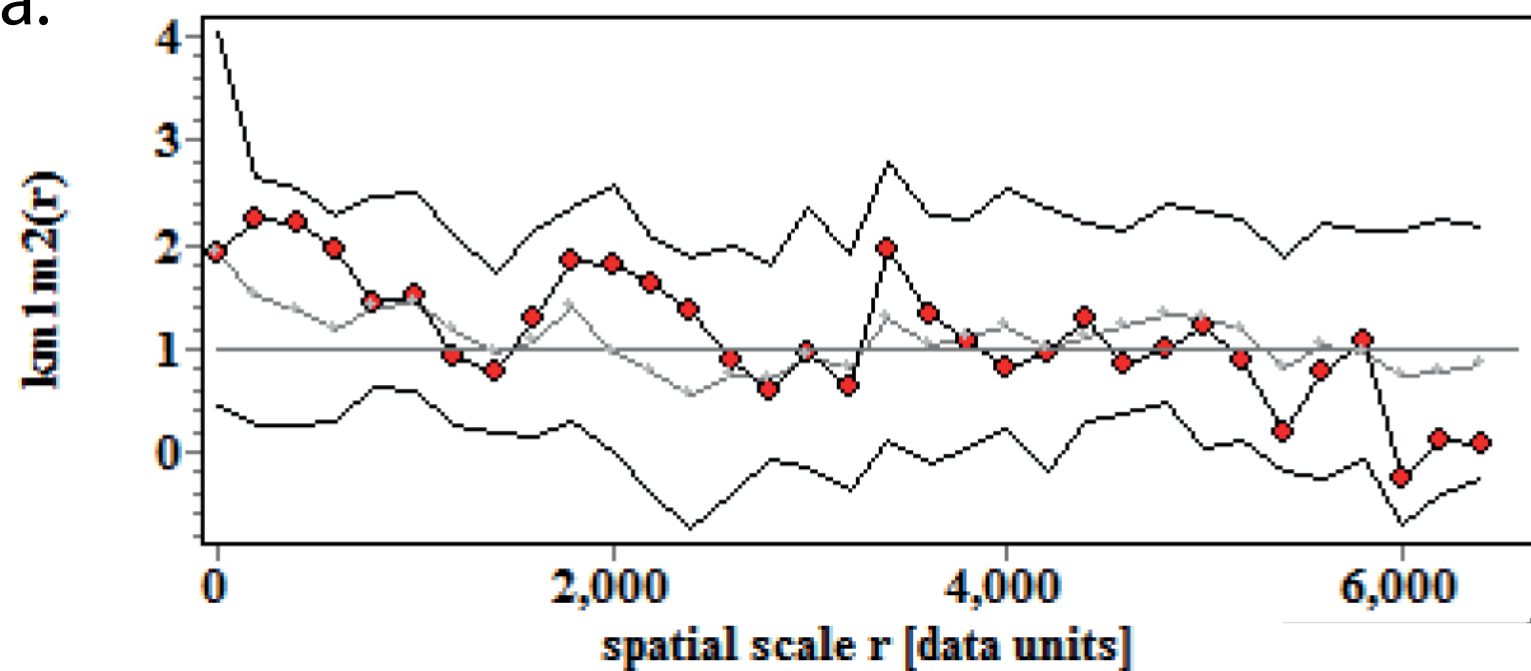

b.

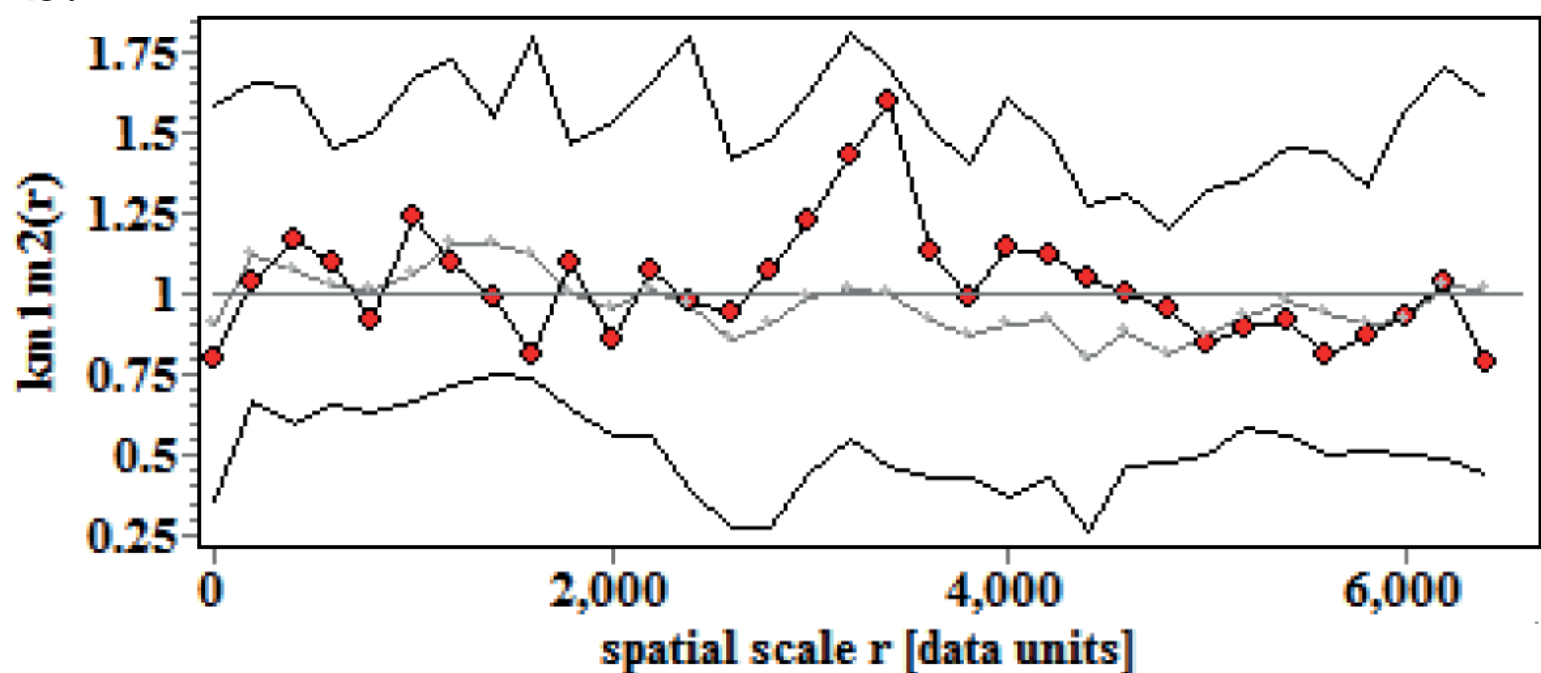

Supplement: Supplementary file 4 — Additional file 4: Figure S2. Bivariate spatial analysis of the relative abundance of T. cruzi-infected T. infestans vs the social vulnerability (a) or host availability indices (b), Area III of Pampa del Indio, Chaco, Argentina. The observed values correspond to the full dark circles and the lines correspond to the expected random pattern and its confidence envelopes. [file 13071_2019_3444_MOESM4_ESM.pdf]
